# Prevalence and characterisation of band-shaped tail lesions in Holstein cows

**DOI:** 10.1186/s13028-024-00742-0

**Published:** 2024-05-14

**Authors:** Tobias Volhøj, Cecilie Kirstine Nielsen, Ditte Marie Schjermer, Natascha Schou Jensen, Benjamin Meyer Jørgensen, Søren Saxmose Nielsen, Henrik Elvang Jensen

**Affiliations:** 1 Vendsyssel Landdyrlaeger, Bredgade 80, Tårs, DK-9830 Denmark; 2 Department of Veterinary and Animal Sciences, Faculty of Health and Medical Sciences, University of Copenhagen, Ridebanevej 3, Frederiksberg C, DK-1870 Denmark

**Keywords:** Cattle, Cow tail, Localisation, Tail wounds

## Abstract

The aim of the study was to characterise and determine the prevalence of band-shaped tail lesions in Holstein cows. Lesions were present either as wounds or by epithelised granulation/connective tissue formations. Both types were characterised by a median localisation 7 cm from the tip of the tail, and they occurred on the dorsal aspect of the tail. From here they encircled the tail either completely or in varying degrees, and they were often present as isolated lesions (93%). The prevalence of band-shaped tail lesions was found to be 25% among 2099 cows examined in 16 Danish Holstein herds with a variation from 18 to 40% between herds. In the herds, the wound lesions and the connective tissue formations accounted for 22% and 78% of all band-shaped tail lesions, respectively. Among 458 Holstein cows examined at an abattoir the prevalence of band-shaped tail lesions was 23%, i.e. similar to the prevalence within the herds. At the abattoir the share of band-shaped wound lesions was 67% and the band-shaped connective tissue formation 33%. Associations between the occurrence of band-shaped tail lesions and parity and lack of the tail tip were observed.

## Findings

In dairy cows, tail lesions such as dermatophilosis, rat-tail syndrome, tail hood, tail thread, tail fractures/dislocations, tail-tip necrosis and band-shaped tail (BST) lesions localised to the skin and underlying structures occur with varying frequencies [1–12]. The BST lesions are a relatively undescribed phenomenon in dairy cows. They are characterised by lesions appearing either as wounds or as band-shaped, more or less hairless, connective tissue formations. The BST lesions appear as isolated or parallel bands that go across the dorsal aspect of the tail, and they are localised distally on the tail, enclosing it to varying degrees. In a German study from 2022, the prevalence of similar shaped lesions, however entitled “ring-like alterations”, was 24% in Holstein cows [1]. Apart from the German report, BST lesions have hitherto not been in focus, therefore, practically all aspects of this type of tail lesions including aetiology, pathogenesis, patho-anatomical characterisation and impact, are unknown. Over the past few years, veterinarians and farmers in northern Jutland, Denmark in particular have been aware of BST lesions in dairy cows and have in some cases associated them with auto-amputation of the distal part of the tail. In other cases, veterinarians have had to amputate the tails due to profound infection in the band-shaped wounds. Based on the observations from northern Jutland and a basic interest in lesion occurrence in our domestic mammals, a study was initiated to characterise these lesions and map the prevalence in herds and in cows received at an abattoir. In the study, we aimed to determine the prevalence of BST lesions in Holstein cows, describe their morphological characteristics, and determine whether they were associated with the presence of tail fractures/dislocations or missing tail tips.

Two sub-studies were carried out, one in an abattoir and one in 16 conventional Danish dairy herds, in the same geographic area of Denmark.

At the abattoir, the animals included were Holstein cows from conventional herds. The full extent of each tail was examined visually and by palpation. Tail fractures/dislocations were recorded. The tails’ hair coat was then clipped and the skin examined for band-shaped lesions defined as wounds or connective tissue formation, and the circumference of the lesions measured (as a percentage of the total tail circumference at the lesion level), as well as whether the tail tip was missing (shortened with wound/scar tissue formation and without hair on the stump). The distance from the centre of the BST lesions to the tail tip was measured with a ruler. All BST lesions were characterised, if a tail had multiple BST lesions, and tissues were sampled for histological examination from both types of the BST lesions. Cross sections of the tails including the lesions and coccygeal vertebrae were fixed in 10% buffered formalin for one week and then decalcified in EDTA for three weeks. Following decalcification, sagittal tissue sections were made and processed for histology by routine methods. The trimming of the lesions was carried out sagittally with respect to the orientation of the tail, i.e. longitudinally.

Herds included were different with respect to management and housing including manure removal systems (manual (*n* = 7), scraper (*n* = 4) or robot-scraper (*n* = 5)), floor types (concrete (*n* = 10) or slatted floors (*n* = 6)), milking systems (tie-stall (*n* = 5), carousel (*n* = 2), milking-parlour (*n* = 5), or automatic milking systems (*n* = 4)), cow cubicle (mattress (*n* = 13), sand (*n* = 3)), housing (tie-stall (*n* = 4) or free range stable (*n* = 12)), and tail-clipping routines (regularly (*n* = 13) or not done (*n* = 3). Only Holstein cows (parity ≥ 1) were included.

In total, 16 herds were selected for the examination of tails. The population of herds was a convenience sample being clients of one of the authors (TV). Within herds, the tails of all cows were examined without regard to lesions. If tail hairs were not regularly clipped, this was done before the examinations, which typically took place in connection with milking. All cows were fixated in catch guards/milking systems, whereas cows standing or lying in beds were examined there. Occurrence of BST lesions and the presence of tail fractures/dislocations were recorded as at the abattoir.

The prevalence of tail fracture/dislocation and BST lesions was determined as the proportion of animals with tail lesions of the total proportion of cows studied. Cross tabulation of the variables studied (parity group (1, 2, 3, and > 3) and tail fracture/dislocation), with and without lesions, was made. Based on these, χ2-tests were used to examine whether there was a difference in the prevalence of the respective variables.

**Fig. 1:**
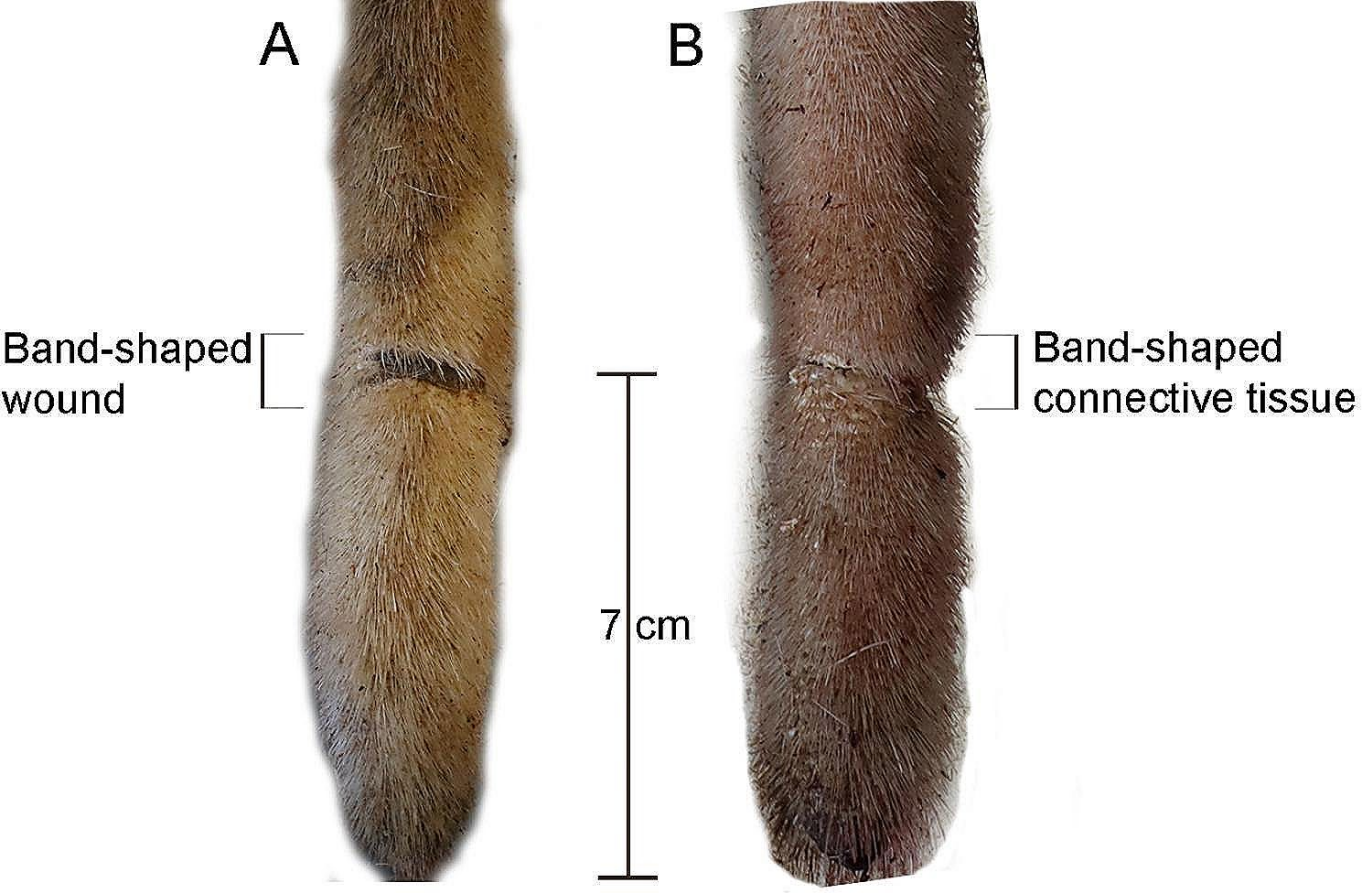
Typical localisation of band-shaped tail wounds (**A**) and connective tissue formations (**B**) in Holstein cows

At the abattoir, 458 Holstein cows from 55 herds, which delivered between 1 and thirteen animals, were examined, and the prevalence of the BST lesions was found to be 23%. Out of the total number of lesions (*n* = 141), BST wounds constituted 67%, and BST connective tissue formation was found in 33%. In the herds, a total of 2099 dairy cows were examined, and a prevalence of 25% of BST lesions was observed. Of the total number of BST lesions in the herds (*n* = 535), the proportion of lesions classified as wounds was 22% while 78% comprised connective tissue formations. In individual herds, the prevalence of BST lesions ranged from 18 to 40%. Both at the abattoir and in herds, the median location of the BST lesions was 7 cm from the tail tip (Fig. 1). On each tail the presence of an isolated lesion was predominant (93%) and always localised on the dorsal aspect of the tail, which they enveloped to varying degrees. At the abattoir, the circumference was found to vary from 41 to 69% of the tail with an average of 46%. Similarly, an average circumference of approximately 50% (ranging from 25 to 100% of the circumference) was found in the herds. In both studies, wounds were found to vary in width (from 0.5 cm up to 3 cm) and depth, from superficial to deep-seated and almost perforating ulcerations leaving only a few soft tissue structures present that kept the tip of the tail still attached to the proximal part. In the distal part of these tails including the tail tip, varying degrees of necrosis/gangrene was present. Therefore, these deep and circumferential ulcerations eventually may lead to auto-amputation of the distal part of the tail. All BST wounds were chronic and apart from crust formation accompanied by suppuration and haemorrhage to a varying extent. The BST connective tissue formations, which also varied in width (from 0.5 cm up to 2.5 cm), were in all cases slightly elevated, faded, had increased texture and varying degrees of hair loss (from thinning to alopecia), which was also the case for the stump of amputated tails. Associations were observed between occurrence of BST lesions and the three variables tail fracture/dislocation, lack of the tail tip and parity group (Table 1).

**Table 1:** Cow-level distribution of band-shaped tail lesions stratified by cow-level variables in 16 Danish dairy herds (*n* = 2099) and at one Danish abattoir in northern Jutland, Denmark

Variable		Lesions				P-value
		Yes		No		
		n	%	n	%	
Tail fracture /dislocation	Yes	42	19	178	81	0.02
	No	493	26	1386	74	
Amputated tail tip	Yes	33	15	183	85	0.0003
	No	502	27	1381	73	
Parity group	1	71	21	268	79	0.002
	2	71	29	171	71	
	3	61	31	133	69	
	> 3	69	35	129	65	

**Fig. 2:**
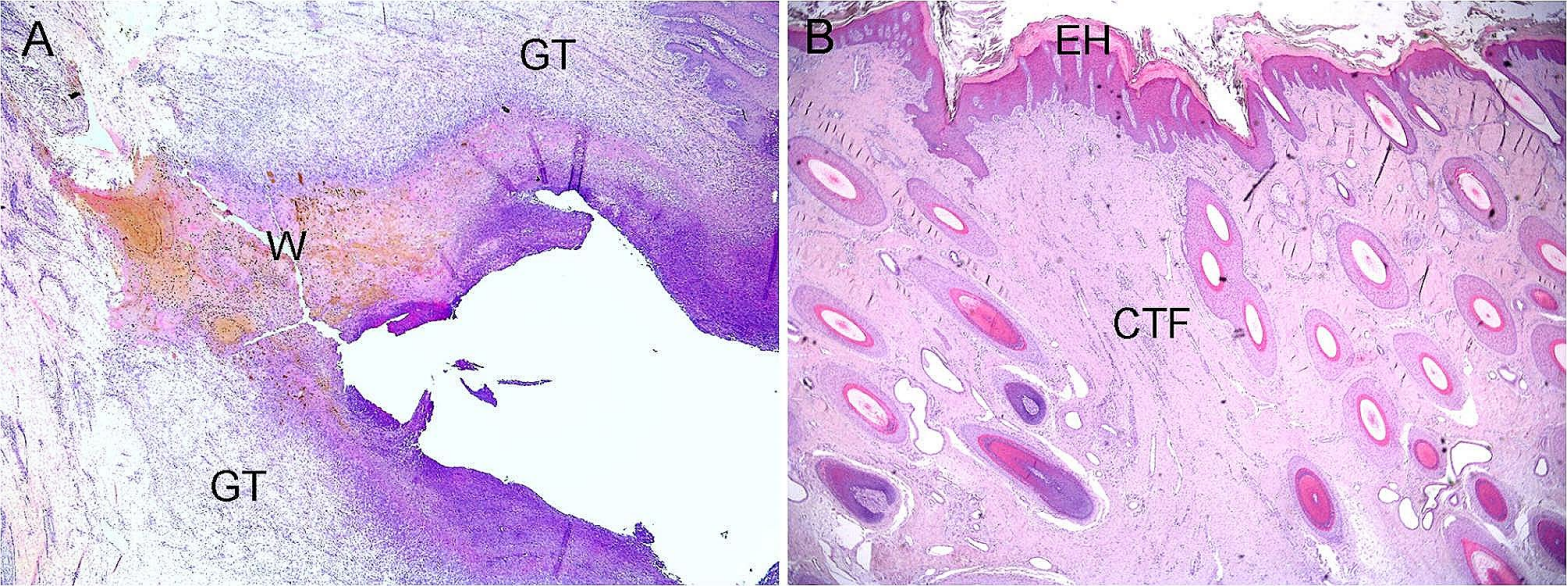
**A**: Histology of a band-shaped tail wound in Holstein cow. Around the haemorrhagic and suppurative wound (W), granulation tissue (GT) with loss of hair follicles and glands is present. **B**: Histology of connective tissue formation (CTF) in a band-shaped pattern on the tail of a Holstein cow. Epidermal hyperkeratosis (EH) with underlying dermal fibrosis and reduced numbers of hair follicles and glands are observed. Haematoxylin and eosin stained sections

The histological examination of the tail wounds revealed that they were healing *per secundam*, characterised by the formation of granulation tissue both at the base and from the sides of the wounds (Fig. 2A). The BST connective tissue formations were characterised by connective tissue formation in the dermal and subcutaneous layers with loss of hair follicles and glands. These areas were covered by epidermal hyperplasia including hyperkeratosis (Fig. 2B).

In both studies, a higher prevalence of BST lesions was observed in higher parity cows, which is consistent with the observations of “ring-like alterations” in a German survey [1]. However, this is not surprising, as older cows will have a longer period at risk. This is also known for several other disorders [13]. However, it might also be relevant to study younger animals, as we in the present study observed several parity 1 cows, which had one of the two types of BST lesions. This could indicate that the condition is present at an earlier age, which could not be determined in the present study due to the inclusion criteria.

It has been hypothesised that BST wounds may lead to auto-amputation of the distal part of the tail. We found a few cases of wounds that fully encircled and almost perforated the tail, leaving the tail tip only attached by tissue remnants. In addition, several tails were found with lesions consistent with a previous auto-amputation. It is important, however, to note that other aetiologies, e.g. tail stumping and other traumatic injuries may be the cause of an equal appearance of the tail tip, and that any missing tail tip therefore is not necessarily a result from BST wound associated auto-amputation. Regardless of the aetiology leading to tail tip amputation these animals are at lower risk of the presence of BST lesions.

Initially, two hypotheses about the association between tail fractures/dislocations and occurrence of BST lesions were made: (1) Tail fractures/dislocations compromise/disturb the blood supply to the tail, which could cause local thrombosis and ischaemic necrosis. (2) Employees’ handling of the animals can result in both lesion types, and that the impact of their handling could be reflected in the prevalence of tail fractures/dislocations [2, 8, 12]. An association was also observed between tail fractures/dislocations, with a higher risk of BST lesions among those without tail fractures/dislocations. This is slightly puzzling, but both types must be considered to be significant welfare issues. The prevalence of tail fractures/dislocation at the abattoir was 16%, while in herds it ranged from 0 to 18.5% with an average of 10.48%. This supports the hypothesis that tail fractures/dislocations are a management problem in herds inflicted by forceful manipulation of the tail by stock people [2, 8, 12].

Although the present study has clarified some of the hitherto unknown conditions regarding the BST lesions in dairy cows, further studies are needed in order to disclose the aetiological and pathogenetic mechanisms that underlie the development of these common and distinct characteristic tail lesions in Holstein cows. In the German study, no genetic background for the origin of the tail lesions entitled “ring-like alterations” was found [14], but with the characteristic segmented shape of the lesions, sometimes with presence of more parallel lesions on the same tail, it is reasonable to believe that the segmented blood supply in the tail [15] could be of pathogenetic significance.

## Data Availability

The datasets used and/or analysed during the current study are available from the corresponding author on reasonable request.
